# Acute coronary syndrome after an infective exacerbation of COPD: a prospective cohort study of acute lower respiratory tract disease in hospitalised adults

**DOI:** 10.1183/23120541.00403-2025

**Published:** 2025-12-15

**Authors:** Caitlin Morgan, Robert Challen, Elizabeth Begier, Jo Southern, George Nava, George Qian, Serena McGuinness, Jade King, Nick Maskell, Maria Lahuerta, Jennifer Oliver, Bradford D. Gessner, Adam Finn, Leon Danon, Catherine Hyams, James W. Dodd

**Affiliations:** 1 Academic Respiratory Unit, Bristol Medical School, University of Bristol and Bristol NIHR Biomedical Research Centre, Bristol, UK; 2 Engineering and Mathematics, University of Bristol, Bristol, UK; 3 Pfizer Vaccines, Collegeville, PA, USA; 4 Bristol Vaccine Centre, Population Health Sciences, University of Bristol, Bristol, UK; 5 Clinical Research and Imaging Centre, University Hospitals Bristol and Weston NHS Trust, Bristol, UK; 6 These authors contributed equally

## Abstract

**Introduction:**

Comorbid cardiovascular disease has been reported extensively in community COPD populations, but to a lesser degree in acute hospital settings. Shared risk factors and acute infection both increase acute coronary syndrome (ACS) risk. Our objective is to assess a cohort of adults hospitalised for an acute lower respiratory tract infection (aLRTI) to determine whether COPD status is an independent risk factor for ACS.

**Methods:**

A prospective observational cohort study of adults aged ≥40 years (n=8496) with community-acquired aLRTI (Bristol, UK) was conducted between 27 July 2020 and 28 November 2022. Cases included physician diagnosis of COPD; controls were aLRTI without COPD. Outcomes included physician-diagnosed ACS occurring within 30 days of admission. Logistic regression models were adjusted for shared cardiovascular risk factors and aLRTI severity.

**Results:**

30-day ACS events in patients hospitalised with aLRTI with COPD were 7.59% (190 out of 2502), *versus* without COPD 6.96% (417 out of 5994) (p=0.3094). Across both groups ACS incidence was 95.1 events per 100 inpatient years. There was no association between COPD and 30-day ACS risk, when adjusting for shared cardiovascular risk factors (OR 1.14, 95% CI 0.93–1.39). However, a diagnosis of COPD increased ACS risk in those without pneumonia *versus* controls (OR 1.38, 95% CI 1.02–1.87). Markers of infection were associated with increased risk of ACS in both groups (white cell count >10×10^9^ cells·L^−1^ OR 1.31, 95% CI 1.10–1.56). Pneumonia was associated with the highest risk of ACS (OR 1.49, 95% CI 1.19–1.87 (no COPD); OR 1.46, 95% CI 1.11–1.92 (with COPD)).

**Conclusions:**

In this large, real-world cohort of hospitalised adults with aLRTI, 30-day ACS event rates were high at ∼7–13%. A diagnosis of COPD even in the absence of pneumonia increases ACS risk *versus* our control group without COPD. Markers of infection severity appear to be key drivers of ACS in this population. This highlights the importance of both COPD and infection severity on risk of ACS following hospitalisation with aLRTI.

## Shareable abstract

**High incidence of ACS following hospitalisation with acute lower respiratory tract infection (aLRTI) in this cohort and the importance of increased vigilance within 30 days of admission with aLRTI regardless of underlying diagnosis of COPD** https://bit.ly/43O2Cab

## Introduction

COPD is a complex heterogeneous condition associated with genetic, environmental and behavioural factors throughout life, including exposure to cigarette smoke and air pollutants [1]. COPD mortality represents 6% of global deaths and has enormous global healthcare costs [2]. Acute worsening of respiratory symptoms (acute exacerbations of COPD (AECOPD)) are commonly caused by lower respiratory tract infections (LRTI) [3]. The overall 5-year survival rate following an AECOPD is <50% [4], and the 90-day readmission rate is 43% [5]. AECOPD is associated with airway and systemic inflammation, accelerated disease progression and a reduction in quality of life [6, 7].

In the COPD population, comorbid cardiovascular disease (ischaemic heart disease (IHD), heart failure, arrhythmias, strokes and disease of pulmonary circulation) is widely recognised. IHD and heart failure are 2.5 times more likely in COPD patients compared to age-matched non-COPD populations [8–11]. Some data suggest that COPD might be an independent cardiovascular disease (CVD) risk factor, after adjusting for confounders [8, 12], and coexisting disease results in worse outcomes than either, in isolation. Complications of CVD are a leading cause of hospitalisations and readmissions in the COPD population, and the second most common cause of death [8, 13–15].

The relationship between CVD and COPD is considered a complex interplay of 1) shared risk factors; 2) shared pro-inflammatory or physiological pathways, particularly during exacerbation; 3) a shared association based on the high global prevalence of both diseases; and 4) side-effects of medications [10]. Older age, smoking, lower socioeconomic background, inactivity, comorbid type two diabetes mellitus, and hypertension are all common risk factors for both CVD and COPD [1, 9, 15–17]. AECOPD is associated with oxidative stress and systemic inflammatory mediator “overspill” contributing to atherosclerotic processes and thrombotic events [3, 15]. Furthermore, the impact of acute LRTI (aLRTI) has also been strongly associated with acute coronary syndrome (ACS) risk in all populations [18].

The available data on incidence and risk of ACS following hospitalised AECOPD is improving [3, 7, 11, 13, 19]. However, there are notable differences in study design, including variation in disease definitions, control populations, underdiagnosis of COPD and CVD, and differing adjustments for smoking and sedentary lifestyle [9, 10, 13, 16, 20]. One of the key limitations is the lack of a well-characterised control group with aLRTI. Therefore, we aim to describe ACS incidence in a hospitalised cohort of patients with aLRTI/AECOPD and determine whether COPD is an independent risk factor for ACS, when compared to patients hospitalised with aLRTI without COPD. This was the first study of its kind to quantify this link and significantly contributes to the discussion about how best to protect patients during acute admissions to hospital. Some of the results of this study have been reported previously in the form of an abstract [21].

## Methods

### Study design

We analysed data from adult hospitalisations (aged ≥40 years) between 27 July 2020 and 28 November 2022, in a prospective observational cohort study undertaking comprehensive surveillance of adults admitted to both acute care hospitals encompassing all admissions in Bristol, UK (AvonCAP, registered at ISRCTN.com, identifier number 17354061). The full study design, including eligibility criteria, have been published previously [22]. The primary objective was to determine whether COPD was an independent risk factor for ACS within 30 days of admission in a hospitalised cohort with aLRTI. The secondary objectives were to calculate the prevalence and risk factors for coexisting CVD in COPD patients and to understand the impact of infection on the risk of ACS in both populations.

Included in this analysis were all aLRTI (controls) and aLRTI with COPD (cases) (table 1). Data on comorbidities were recorded at admission and included the Charlson Comorbidity Index (CCI) [24] (adjusted downwards for COPD patients). We examined the associations between COPD and a clinical diagnosis of acute coronary syndrome (ACS) (table 1). All outcomes, including death, were captured at 30 days following admission, irrespective of discharge from hospital. Severe acute respiratory syndrome coronavirus 2 (SARS-CoV-2) cases exhibited unusual coagulopathy and associated ACS. Because of this uncertainty we restricted our analysis to those patients who tested negative for SARS-CoV-2 infection.

**TABLE 1:** Case definitions

**COPD**	Pre-existing COPD diagnosis at the time of admission was based on documented standard-of-care past medical history
**ACS**	Physician-diagnosed STEMI, non-STEMI and acute left bundle branch block which occurred within 30 days of a participant's admission. Events were captured irrespective of discharge from hospital Diagnosis was determined in accordance with local and national guidelines with clinical features, markers of disease activity (dynamic or persistent troponin I and ECG changes), and imaging (CT and chest radiography)
**SARS-CoV-2**	Respiratory symptoms associated with confirmed SARS-CoV-2 infection
**aLRTI with a background of COPD**	First admission captured by AvonCAP study Sustained symptom worsening from usual stable COPD individual state (beyond normal day-to-day variations) which was acute (<28 days) in onset [23] aLRTI for the purposes of this analysis was caused by infection defined by either a confirmed radiological or microbiological diagnosis of acute respiratory infection or diagnosed by the attending physician on admission
**aLRTI without COPD (control)**	First admission captured by AvonCAP study Diagnosis on admission by the attending physician: 1) Pneumonia: respiratory infection confirmed by pneumonic changes on imaging (CT or chest radiograph reported by a radiologist within 96 h of admission) 2) Non-pneumonic: respiratory infection confirmed by raised inflammatory markers and clinical suspicion without radiological changes
**Noninfective aLRTD**	aLRTD causing respiratory symptoms and signs resulting in admission to hospital not caused by infection, including heart failure

ACS: acute coronary syndrome; SARS-CoV-2: severe acute respiratory syndrome coronavirus 2; aLRTI: acute lower respiratory tract infection; aLRTD: acute lower respiratory tract disease; STEMI: ST elevation myocardial infarction; CT: computed tomography.

The study was approved by the Health Research Authority research ethics committee (REC) East of England, Essex, REC reference 20/EE/0157.

### Statistical analysis

Categorical values were compared using Fisher's exact test, or Chi-squared test for trend in proportions if ordered, and continuous variables with a two-sample Kolmogorov–Smirnov test. Categorical data are presented as counts and percentages, and continuous data as median (interquartile range). Normality of distributions were determined using Anderson–Darling test. Missing values were identified. Additional detail on missing values is provided in the supplementary material and table S1.

Multivariable analysis between the binary variable of ACS and covariates using logistic regression was conducted. We investigated the association between ACS, COPD status, aLRTI severity indicators, including pneumonic changes on imaging, CURB-65 score (confusion, urea >7 mmol·L^−1^, respiratory rate ≥30 breaths·min^−1^, low blood pressure and age ≥65 years), white cell count (WCC) (>10×10^9^ cells·L^−1^) and C-reactive protein levels (>50 mg·L^−1^), and a range of variables selected *a priori* as known CVD risk factors (age, sex, CCI category, Index of Multiple Deprivation (decile), smoking status and past medical history of hypertension, diabetes, peripheral vascular disease and chronic kidney disease). Odds ratios with 95% confidence intervals were determined for each variable in a set of unadjusted models. The CURB-65 score was adjusted to avoid representing age twice in these models.

### Sensitivity analyses

We tested independence of our results to the choice of statistical model by a sensitivity analysis calculating risk ratios using quasi-Poisson regression instead of logistic regression. We performed a sensitivity analyses on measurement of elevation of high-sensitivity cardiac troponin in both groups. The first troponin measured following admission was used for the analysis. If the troponin was part of multiple tests, then the highest was recorded. We assessed the impact of asthma on our results and compared the rates of community-prescribed cardiac medications (supplementary methods).

In all analyses, p-values were interpreted using a level of statistical significance adjusted to account for multiple testing, and adjusted levels of significance presented alongside the results. Statistical analyses were conducted using R version 4.3.1 [25].

## Results

**FIGURE 1:**
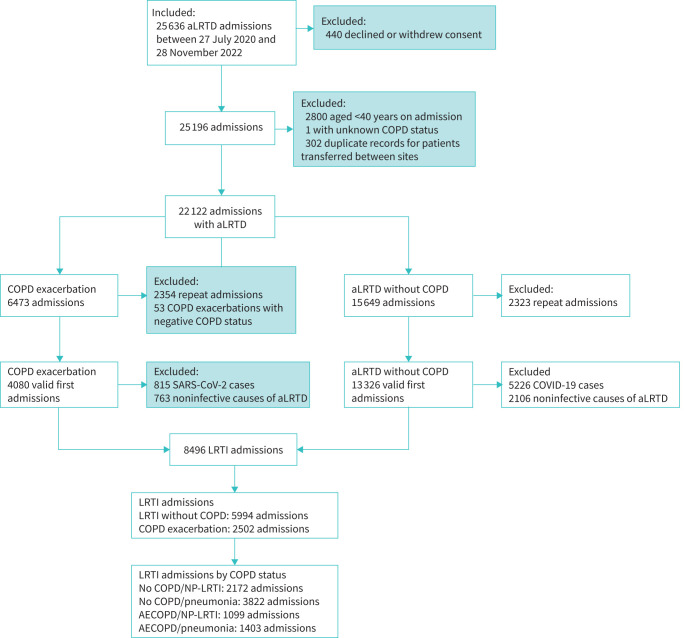
Data flow diagram. Individuals may be excluded for more than one reason; the total number of excluded patients at each stage is not necessarily the sum of the patients excluded for each reason. aLRTD: acute lower respiratory tract disease; SARS-CoV-2: severe acute respiratory syndrome coronavirus 2; LRTI: lower respiratory tract infection; NP: non-pneumonic; AECOPD: acute exacerbation of COPD.

During the study period, 25 636 hospitalisations with acute lower respiratory tract disease (aLRTD) occurred. From these we excluded duplicates, repeat admissions, non infective aLRTD and cases proven to be due to SARS-CoV-2 (figure 1). This resulted in 2502 non-SARS-CoV-2 admissions with infective exacerbation of COPD and 5994 non-SARS-CoV-2 aLRTI admissions without COPD.

Compared to patients admitted with aLRTI without COPD, COPD admissions were significantly younger (age 74.9 years (COPD) *versus* 77 years (no COPD); p<0.001), more frequently current smokers (23.8%, 596 out of 2502 (COPD) *versus* 7.1%, 425 out of 5994 (no COPD); p<0.001) and more frequently presented with elevated white cell count (63.8%, 1597 out of 2502 (COPD) *versus* 56.4%, 3383 out of 5994 (no COPD); p<0.001) (table 2). There were fewer COPD cases, *versus* those without COPD, who presented with pneumonia (56.1%, 1403 out of 2502 (COPD) *versus* 63.8%, 3822 out of 5994 (no COPD); p<0.001).

**TABLE 2:** Demographic characteristics of the study cohort stratified by clinical presentation of acute lower respiratory tract infection (LRTI)

	LRTI without COPD	LRTI with COPD	p-value
**Participants**	5994	2502	
**aLTRD presentation**			
Pneumonia	63.8 (3822)	56.1 (1403)	<0.001^#^
NP-LRTI	36.2 (2172)	43.9 (1099)	
**Age-adjusted CURB category**			
0–1 (mild)	93.2 (5589)	94.4 (2363)	0.062^¶^
2 (moderate)	6.3 (376)	5.1 (127)	
3–5 (severe)	0.5 (29)	0.5 (12)	
**Age years**	77 (63.3–86.1)	74.9 (66.6–81.9)	<0.001^+^
**Gender**			
Female	49.7 (2980)	49.0 (1227)	0.58^#^
Male	50.3 (3014)	51.0 (1275)	
**Ethnicity**			
White British	76.9 (4611)	83.5 (2089)	<0.001^#^
White other	1.9 (115)	1.5 (37)	
Mixed origin	0.6 (38)	0.5 (13)	
Black	1.6 (95)	0.7 (18)	
Asian	1.6 (93)	0.4 (11)	
Other	0.6 (37)	0.4 (10)	
Unknown	16.7 (1003)	12.9 (324)	
Missing	0.0 (2)	0.0 (0)	
**CCI category excluding COPD**			
None (0)	5.9 (354)	2.2 (56)	<0.001^#^
Mild (1–2)	17.7 (1060)	17.5 (439)	
Moderate (3–4)	31.8 (1909)	40.6 (1017)	
Severe (≥5)	44.5 (2668)	39.6 (990)	
Missing	0.1 (3)	0.0 (0)	
**IMD (decile)**	6 (3–8)	5 (3–7)	^§^
**Smoking status**			
Nonsmoker	44.6 (2672)	6.0 (151)	<0.001^#^
Current smoker	7.1 (425)	23.8 (596)	
Ex-smoker	38.0 (2275)	64.3 (1610)	
Unknown	10.4 (622)	5.8 (145)	
**Hypertension**	14.7 (882)	14.6 (365)	0.89^#^
**Atrial fibrillation**	18.6 (1112)	16.5 (412)	0.024^#^
**CVA/TIA**	10.9 (652)	11.6 (291)	0.32^#^
**IHD**	13.4 (803)	15.3 (382)	0.026^#^
**CCF**	13.4 (803)	14.1 (354)	0.37^#^
**Diabetes**			
None	79.1 (4742)	78.8 (1972)	0.07^#^
Type 1	1.3 (78)	0.8 (19)	
Type 2	19.6 (1174)	20.4 (511)	
**Peripheral vascular disease**	2.9 (172)	4.6 (116)	<0.001^#^
**CKD**			
None	73.5 (4407)	76.1 (1905)	0.036^#^
Mild (CKD 1–3)	22.3 (1336)	20.3 (509)	
Moderate or severe CKD (CKD ≥4)	4.2 (251)	3.5 (88)	
**Troponin** **level**			
≤18 ng·L^−1^	16.7 (1001)	14.2 (356)	0.0017^#^
>18 ng·L^−1^	22.6 (1353)	21.2 (530)	
Unknown	60.7 (3640)	64.6 (1616)	
**CRP level**			
<10 mg·L^−1^	19.4 (1160)	21.9 (547)	0.0038^#^
10–50 mg·L^−1^	30.7 (1842)	31.9 (798)	
>50 mg·L^−1^	48.8 (2923)	45.5 (1139)	
Unknown	1.2 (69)	0.7 (18)	
**White cell count**			
≤10×10^9^ cells·L^−1^	43.0 (2577)	35.7 (893)	<0.001^#^
>10×10^9^ cells·L^−1^	56.4 (3383)	63.8 (1597)	
Unknown	0.6 (34)	0.5 (12)	
**Death within 30 days**	13.4 (802)	11.8 (295)	0.047^#^

Data are presented as n, % (n), median (interquartile range), unless otherwise stated. Normal distributions were determined using the Anderson–Darling test (p>0.005). An adjusted p-value of 0.0025 was considered significant. aLRTD: acute lower respiratory tract disease; NP: non-pneumonic; CURB-65: confusion, urea >7 mmol·L^−1^, respiratory rate ≥30 breaths·min^−1^, low blood pressure and age ≥65 years; CCI: Charlson Comorbidity Index; IMD: Index of Multiple Deprivation; CVA: cerebrovascular accident; TIA: transient ischaemic attack; IHD: ischaemic heart disease; CCF: congestive cardiac failure; CKD: chronic kidney disease; CRP: C-reactive protein. ^#^: Fisher's exact test (categorical); ^¶^: Chi-squared test for trend in proportions (ordered); ^+^: two-sample Kolmogorov–Smirnov test (continuous); ^§^: not calculated due to missing values (continuous).

### Primary objective

The overall incidence of 30-day ACS in the study population was 607 (7.14%) out of 8496 and did not significantly vary between those with and without COPD (table 3). Unadjusted comparisons using logistic regression (table 4) showed significant correlation between an ACS outcome and pneumonia, CURB-65 score ≥2, patient age at admission, CCI ≥1, IHD and an elevated admission WCC. In adjusted model 1, we continued to see association of age with history of IHD (table 4) and ACS. Adjusted model 2 (table 4) found that several factors including pneumonic changes on imaging, elevated CURB-65 score and CCI category, history of IHD and elevated WCC were significantly associated with increased ACS risk in hospitalised aLRTI. Further analysis showed that in the groups without pneumonic changes on imaging, COPD was associated with increased risk of ACS (OR 1.38, 95% CI 1.02–1.87). Pneumonia in both groups was associated with the highest risk of ACS (OR 1.49, 95% CI 1.19–1.87 (no COPD); OR 1.46, 95% CI 1.11–1.92 (with COPD)). In a higher risk subgroup including patients aged >65 years, elevated WCC, IHD, and with pneumonia, the ACS rate was 13.24% (53 out of 381) compared to 7.14% (607 out of 8496) for the whole cohort.

**TABLE 3:** Rate of acute coronary syndrome (ACS) in the study population

	ACS rate	Follow-up years	Incidence per 100 admitted patient years
**All LRTI admissions excluding SARS-CoV-2**	607/8496 (7.14)	638.33	95.1
**LRTI without COPD**	417/5994 (6.96)	447.91	93.1
NP-LRTI	110/2172 (5.06)	171.51	64.1
Pneumonia	307/3822 (8.03)	276.40	111.1
**COPD exacerbation**	190/2502 (7.59)	190.42	99.8
NP-LRTI	78/1099 (7.10)	87.19	89.5
Pneumonia	112/1403 (7.98)	103.23	108.5

Data are presented as n/N (%) unless otherwise stated. Follow-up was calculated based on the 30-day outcome adjusting for those who died prior to this. LRTI: lower respiratory tract infection; SARS-CoV-2: severe acute respiratory syndrome coronavirus 2; NP: non-pneumonic.

**TABLE 4:** Logistic regression for the association between different covariates and a clinical diagnosis of acute coronary syndrome (ACS) during an inpatient admission.

	Unadjusted^#^		Adjusted model 1^¶^		Adjusted model 2^+^	
	OR (95% CI)	p-value	OR (95% CI)	p-value	OR (95% CI)	p-value
**COPD LRTI status**						
LRTI without COPD	Ref.	0.301	Ref.	0.201		
COPD exacerbation	1.10 (0.92–1.31)		1.14 (0.93–1.39)			
**COPD pneumonia interaction**						
No COPD/NP-LRTI	Ref.	<0.001			Ref.	0.004
No COPD/pneumonia	1.64 (1.31–2.05)				1.49 (1.19–1.87)	
COPD/NP-LRTI	1.43 (1.06–1.93)				1.38 (1.02–1.87)	
COPD/pneumonia	1.63 (1.24–2.13)				1.46 (1.11–1.92)	
**Age-adjusted CURB category**						
0–1 (mild)	Ref.	0.004			Ref.	0.045
2 (moderate)	1.59 (1.18–2.14)				1.42 (1.05–1.92)	
3–5 (severe)	2.32 (0.97–5.53)				1.83 (0.76–4.39)	
**Age in decades**	1.18 (1.11–1.25)	<0.001	1.15 (1.07–1.23)	<0.001	1.05 (0.95–1.15)	0.334
**Gender**						
Female	Ref.	0.700				
Male	1.03 (0.88–1.22)					
**CCI category excluding COPD**						
None (0)	Ref.	<0.001			Ref.	0.032
Mild (1–2)	2.04 (1.08–3.88)				1.75 (0.91–3.38)	
Moderate (3–4)	2.49 (1.34–4.62)				1.79 (0.90–3.57)	
Severe (≥5)	3.57 (1.94–6.57)				2.30 (1.13–4.68)	
**IMD (decile)**	1.00 (0.97–1.02)	0.767				
**Smoking status**						
Nonsmoker	Ref.	0.059	Ref.	0.157		
Current smoker	0.87 (0.64–1.17)		0.95 (0.68–1.31)			
Ex-smoker	1.07 (0.88–1.29)		0.98 (0.80–1.20)			
Unknown	1.39 (1.05–1.85)		1.34 (1.01–1.79)			
**Hypertension**	1.24 (0.99–1.54)	0.064	1.00 (0.79–1.25)	0.981		
**Atrial fibrillation**	0.98 (0.79–1.21)	0.836				
**CVA/TIA**	1.08 (0.84–1.40)	0.539				
**IHD**	1.82 (1.49–2.23)	<0.001	1.66 (1.34–2.05)	<0.001	1.60 (1.29–1.98)	<0.001
**CCF**	1.31 (1.05–1.63)	0.021	1.08 (0.85–1.36)	0.534	1.02 (0.81–1.30)	0.848
**Diabetes type**						
None	Ref.	0.709	Ref.	0.846		
Type 1	1.19 (0.57–2.47)		1.24 (0.59–2.59)			
Type 2	1.08 (0.88–1.32)		1.02 (0.83–1.26)			
**Peripheral vascular disease**	1.25 (0.82–1.89)	0.318				
**CKD**						
None	Ref.	0.705				
Mild (CKD 1–3)	1.07 (0.88–1.31)					
Moderate or severe CKD (CKD ≥4)	0.91 (0.59–1.42)					
**CRP level**						
Unknown	0.72 (0.26–2.00)	0.298				
<10 mg·L^−1^	Ref.					
10–50 mg·L^−1^	1.19 (0.93–1.52)					
>50 mg·L^−1^	1.20 (0.95–1.50)					
**White cell count**						
Unknown	0.35 (0.05–2.55)	<0.001			0.38 (0.05–2.81)	0.004
≤10×10^9^ cells·L^−1^	Ref.				Ref.	
>10×10^9^ cells·L^−1^	1.37 (1.15–1.63)				1.31 (1.10–1.56)	

Due to there being 18 comparisons in the unadjusted column statistical significance should be considered at a level of p<0.003. In the adjusted models, a level of p<0.05 is appropriate as each adjusted model can be regarded as a single comparison. LRTI: lower respiratory tract infection; NP: non-pneumonic; CURB-65: confusion, urea >7 mmol·L^−1^, respiratory rate ≥30 breaths·min^−1^, low blood pressure and age ≥65 years; CCI: Charlson Comorbidity Index; IMD: Index of Multiple Deprivation; CVA: cerebrovascular accident; TIA: transient ischaemic attack; IHD: ischaemic heart disease; CCF: congestive cardiac failure; CKD: chronic kidney disease; CRP: C-reactive protein; Ref.: reference. **^#^**: unadjusted univariate models; ^¶^: a combination of cardiac risk factors, to adjust ACS risk for background COPD; **^+^**: a combination of factors associated with LRTI severity including pneumonic changes on imaging, and a minimal set of cardiac comorbidities.

### Secondary and sensitivity analyses

In a subanalysis of patients with complete medication history, statin and antiplatelet agent prescription rates were not significantly different in patients with or without COPD. However, β-blockers were less commonly prescribed in those with COPD than those without (8.1% (16 out of 198) (COPD) *versus* 38.6% (78 out of 202) (no COPD); p≤0.001) (table 5), despite similar CVD prevalence between groups. Respiratory conditions noted in the non-COPD group included asthma and cystic fibrosis. There were more patients with bronchiectasis in the COPD group (supplementary table S2). Sensitivity analysis excluding asthmatics did not change the overall analysis results (supplementary table S3). In a subanalysis of complete data, a trend to increased readmission within 90 days secondary to cardiac complications was seen in COPD patients (36.4% (72 out of 198) (COPD) *versus* 27.2% (55 out of 202) (no COPD); p=0.054).

**TABLE 5:** Comparison of secondary prevention measures between a matched cohort of acute lower respiratory tract infection (aLRTI) patients without COPD and with COPD (matched on age, approximate admission date and gender)

	COPD exacerbation	aLRTI without COPD	p-value^#^
**Participants**	198	202	
**No heart diagnosis**	55.6 (110)	55.4 (112)	1
**CCF**	17.2 (34)	13.9 (28)	0.41
**IHD**	18.2 (36)	16.3 (33)	0.69
**Hypertension**	11.6 (23)	12.9 (26)	0.76
**Atrial fibrillation**	17.7 (35)	18.3 (37)	0.9
**Other arrythmia**	3.0 (6)	1.0 (2)	0.17
**Pacemaker**	3.0 (6)	5.4 (11)	0.32
**Valvular heart disease**	2.0 (4)	3.5 (7)	0.54
**Antiplatelet treatment**	38.4 (76)	41.1 (83)	0.61
**Statin**	31.3 (62)	39.6 (80)	0.095
**β-blocker**	8.1 (16)	38.6 (78)	<0.001
**90-day readmission all-cause cardiac**	36.4 (72)	27.2 (55)	0.054

Data are presented as n or % (n), unless otherwise stated. CCF: congestive cardiac failure; IHD: ischaemic heart disease. ^#^: Fisher's exact test (categorical).

Troponin requests on admission were used as a proxy for assessing clinical suspicion of ACS in both groups (supplementary table S4). 35% (886 out of 2502) (COPD) and 39% (2354 out of 5994) (no COPD) of patients had a troponin test sent. Admission troponin levels did not differ significantly between both cohorts in those with confirmed ACS and those without. A sensitivity analysis using Poisson regression to identify ACS risk ratios generated to similar results to the initial analysis (supplementary tables S5–S7), suggesting that these results were not reliant on the choice of statistical modelling.

## Discussion

This novel analysis of hospitalised COPD patients from a large prospective real-world observational study of hospitalised patients with acute lower respiratory tract infections was designed to explore the impact on risk of ACS in the first 30 days following hospital admission. Our analysis showed high rates of 30-day ACS and supports the finding that a history of COPD on admission increases ACS risk in the absence of pneumonia. Infection severity including pneumonia on imaging and comorbid CVD pose the greatest risk for ACS.

Coexisting CVD in the COPD patient is well recognised, but there is conflicting evidence as to whether ACS risk is due to acute exacerbations of COPD. This analysis provides an accurate assessment of ACS incidence in a hospitalised COPD population with acute infective exacerbation, and comparison with a novel and well characterised control group of hospitalised patients with aLRTI without COPD. The ACS incidence within the first 30 days of admission was broadly similar in both groups (7.59% *versus* 6.96%, respectively, with incidence rate of 99 (COPD) and 93 (no COPD) per 100 inpatient-days. While in an adjusted model COPD was not identified as an independent risk factor for post-hospitalisation ACS, markers of infection, such as pneumonic changes on imaging, CURB-65 score and elevated WCC, increased ACS risk in both COPD and control subjects. Adjusted model 2 did show that COPD raised the risk of ACS in the context of non-pneumonic LRTI (OR 1.38, 95% CI 1.02–1.87), and pneumonia raises risk (approximate OR 1.46/1.49 with/without COPD), but the combination of COPD and pneumonia was no worse than pneumonia without COPD. Contrary to the overall model, these findings may reflect a selection bias based on the criteria by which we image our patients with COPD.

The majority of acute exacerbations of COPD cases are caused by aLRTI [3]. Pro-inflammatory mediators and markers of systemic inflammation, which upregulate cytokine pathways and are pro-thrombotic, are commonly elevated in noninfective exacerbations of COPD, infection-associated COPD and aLRTI in patients without COPD. Studies have confirmed a significant association between infection with common respiratory viruses (influenza and respiratory syncytial virus) and both bacterial pneumonia and myocardial infarction (MI). This risk is higher at onset of infection and directly correlated with infection severity [18, 26, 27]. Our analysis supports the hypothesis that infection severity and concurrent CVD has a greater impact on ACS risk than a background of COPD alone [28].

Coexistence of CVD and shared risk factors are thought to be the most important factors increasing the risk of ACS in COPD patients [10, 15]. When compared to our control group of hospitalised adults without COPD, COPD patients were younger, had a higher prevalence of CCF, IHD, stroke, hypertension, type 2 diabetes mellitus and were more likely to be ex- or current smokers. The CVD prevalence in both populations was consistent with the literature, with the exception of a higher prevalence of atrial fibrillation seen in patients without COPD in other datasets [9, 10, 29]. Other analyses found that pre-existing CVD significantly increased MI risk in COPD. However, incomplete data on smoking history has often made it difficult to draw firm conclusions. In this cohort, we had smoking status data in all but 5.8% (145 out of 2502) and 10.4% (622 out of 6961) of those with and without COPD, respectively.

Inconsistency in incidence and prevalence of CVD in COPD can be partly explained by underdiagnosis and inconsistent treatment of COPD [13, 30]. However, overlapping clinical presentations, poorer performance status and evidence that biomarkers used for detection of cardiac strain (for instance, troponin) are elevated at baseline may also account for underdiagnosis of CVD in COPD [6, 10, 31]. The UPLIFT trial performed a within person assessment of risk and showed an increased risk of MI in the 30 days following an exacerbation of COPD when compared with the 30 days before (incidence rate ratio 13.04, 95% CI 1.71–99.7) [7]. The SUMMIT trial [11] also performed *post hoc* cohort analysis on adverse events, concluding that exacerbations of COPD were associated with increased risk of MI (HR 7.0, 95% CI 4.0–12.3). A recent large cohort study by Graul *et al.* [19] explored the impact of moderate and severe exacerbations on nonfatal cardiovascular events and were able to determine that roughly 28 per 100 hospitalisations with AECOPD will suffer a cardiovascular event. The findings of Graul *et al.* [19] contributed to the updated 2025 Global Initiative for Chronic Obstructive Lung Disease (GOLD) recommendations which increase awareness of cardiovascular events following COPD exacerbation [32]. Our study captures ACS outcomes in the first 30 days following admission. The evidence suggests that the first 30 days following an exacerbation carries the most risk for subsequent ACS, and therefore supports the time frame of this analysis. Comparison of hospitalised AECOPD with nonhospitalised AECOPD showed that CVD events in the first 30 days following admission occurred at almost double the rate in hospitalised exacerbations (26.7 per 100 patient-years) [11]. This is similar to the 2.27-fold (95% CI 1.10–4.70) increased risk of MI in the first 5 days after exacerbation found by Donaldson *et al.* [3] and the 9.9-fold (95% CI 5.0–19.4) increased risk in the first 7 days by Swart *et al.* [33], both in community, nonhospitalised COPD populations. Direct comparison of these findings with our analysis is limited. The incidence of ACS per 100 patient-years in our AECOPD group is 3.4 times higher than that seen in the hospitalised SUMMIT subanalysis. Both COPD cases and controls in our real-world hospitalised cohort analysis were, on average, 10 years older that the SUMMIT trial, which comprised a healthy population (albeit with CVD or risk of CVD) eligible for a lengthy study. Other key differences include determining risk by within-person assessment from baseline, and only included current or former smokers, with moderate–severe airflow obstruction. Furthermore, the SUMMIT population had CVD or were otherwise deemed at increased CVD risk. In contrast, Graul *et al.* [19] assessed risk of hospitalisation with ACS following a community or hospitalised AECOPD. As expected, the cardiovascular event rate in the severe AECOPD group (hospitalised) was higher than the community group. The Graul analysis had a higher mean age, a greater proportion of current smokers and included both infective and noninfective AECOPD. However, despite the differences in methodology, the ACS event rate in the hospitalised exacerbation group in the Graul *et al.* [19] study is similar to our analysis (8.4% *versus* 7.59%).

Our study had many strengths and limitations, which have been discussed previously [22, 34]. The study design ensured full case ascertainment, reducing bias by minimising exclusion of vulnerable and hard-to-reach groups. However, this was a single-centre study and participants were principally Caucasian, which reduces generalisability. The AvonCAP data collection period included the SARS-CoV-2 pandemic, which has been shown to reduce overall COPD admissions [35], which may have affected our findings. The health-seeking behaviour of patients during the pandemic may have resulted in a greater severity of infection in both the control and COPD groups. However, the epidemiology of respiratory infection continuously evolves with time and location which, in this instance, included the SARS-CoV-2 pandemic. The large patient numbers in the cohort and the real-world setting provide assurance that the analyses undertaken were representative of the risk to those admitted with and acute infective exacerbation of COPD during the period studied. Background COPD status was based on physician diagnosis (in primary or secondary care) noted as part of a patient's medical history taken on admission to hospital. The accuracy of primary care diagnosed COPD has been evaluated in the literature as reliable since incentivised registers were introduced in 2004, even in the absence of documented spirometry [36]. This may have led to an under- or over-representation of COPD patients in either group given the frequency of current or ex-smokers, aged >40 years, in the non-COPD group. The magnitude and direction of the effect is uncertain, but it may explain the negative findings of this study. Importantly, this study was a real-world analysis of patients with a diagnosis of COPD on treatment compared to those without a diagnosis and therefore, who are not on treatment. We were unable to determine the baseline respiratory function or therapy, including long- and short-acting bronchodilators, of COPD patients in this cohort. Consequently, we could not determine and adjust by COPD severity scores such as GOLD criteria or use of oral corticosteroids, which have been shown to further increase risk of MI [37]. We are also not able to draw conclusions about the independent risk of COPD in community populations with less severe exacerbations or aLRTI. Our ACS cases were not independently adjudicated. Further work on verifying diagnosis would have added weight the discussion regarding under- or over-diagnosis of ACS in these two populations and would have allowed us to draw more definitive conclusions regarding the use of biomarkers such as troponin. The sensitivity analysis included a comparison of troponin measurements requested between COPD and controls in order to detect systematic differences clinical assessment of ACS, a more detailed analyses of outcomes and characteristics of subgroups with positive or negative troponin were beyond the scope of this analysis. Furthermore, the assessment of other potential biomarkers of increased ACS risk such as blood eosinophil count were not available in the selected cohort for this analysis. Only infective exacerbations of COPD were included. This allowed direct comparisons to our control group, but also allowed for this subgroup of AECOPD to be analysed independently. The cohort only included patients with acute respiratory infection; thus, comparisons of CVD risk in patients admitted with other infections could not be undertaken. Finally, this analysis did not include readmissions in either group. There is evidence that the more frequent exacerbator phenotype is associated with worse risk of ACS *via* increased use of oral corticosteroids and lower forced expiratory volume in 1 s [3, 7, 11, 38]. The assessment of readmissions is an area for future study within this cohort.

In conclusion, in this large, real-world cohort of hospitalised adults with aLRTI, 30-day ACS event rates were high, ranging from 7.1% to 13.2% in certain high-risk groups. We found that a clinical diagnosis of COPD even in the absence of pneumonia increases ACS risk in comparison to our control group without COPD. Furthermore, pre-existing cardiovascular disease and markers of infection severity are key risk factors for subsequent ACS in this hospitalised population. Supplementary analysis has supported the theory that COPD patients are under treated with secondary cardiac prevention, such as β-blockers. We found no evidence that there is a systematic underdiagnosing of ACS in COPD, as we noted similar frequencies of troponin requests across the two groups.

Analysis of this novel, well-phenotyped hospitalised cohort has provided detailed insights into the increased risk of ACS associated with pre-existing COPD and more severe lower respiratory tract infection. If we are to reduce the rate of ACS following hospitalisation with aLRTI, future work should look to mitigate these risk factors with targeted, early treatment and evidenced based secondary prevention.

## Data Availability

The data used in this study are sensitive and cannot be made publicly available without breaching patient confidentiality rules. Therefore, individual participant data and a data dictionary are not available to other researchers.

## Supplementary material

**Please note:** supplementary material is not edited by the Editorial Office, and is uploaded as it has been supplied by the author. Supplementary material 00403-2025.SUPPLEMENT
