# Supplementary material for: Acute coronary syndrome after an infective exacerbation of COPD: a prospective cohort study of acute lower respiratory tract disease in hospitalised adults
Source: ERJ Open Res. 2025 Dec 15;11(6):00403-2025. doi: 10.1183/23120541.00403-2025 (PMC12704152; doi:10.1183/23120541.00403-2025)
Supplement: Supplementary file 1 [file 00403-2025.SUPPLEMENT.pdf]

ONLINE DATA SUPPLEMENT

Acute coronary syndrome after an infective exacerbation of chronic obstructive pulmonary disease (COPD). A prospective cohort study of acute lower respiratory tract disease in hospitalised adults.

Dr Caitlin Morgan[†], Dr Robert Challen[†], Dr Elizabeth Begier, Dr Jo Southern, Dr George Nava, Dr George Qian, Ms Serena McGuinness, Ms Jade King, Dr Maria Lahuerta, Prof Nick Maskell, Dr Jennifer Oliver, Dr Bradford D. Gessner, Professor Adam Finn, Professor Leon Danon, Dr Catherine Hyams[†], Professor James Dodd PhD, The Avon CAP Research Group

METHODS

Missing Data

Where missing values were proven to be missing at random at <10%, values were imputed using chained equations with the R package mice, and all subsequent analysis performed on 10 imputed data sets in parallel. Results were combined as a mixture distribution of the beta coefficients arising from the parallel analyses, assuming that they are normally distributed.

Table S1: Missing data summary for the a-priori variables hypothesised to be related to cardiovascular outcomes in COPD patients with an acute exacerbation.

Considered are age, gender, ethnicity, body mass index (BMI), Charleson comorbidity index (CCI) excluding COPD score, Index of multuple deprivation (IMD) decile, smoking status, pre-existing hypertension, diabetes, peripheral vascular disease, chronic kidney disease (CKD) and acute lower respiratory tract disease admission category (COVID infection, non COVID infection, and non infective). Data was unavailable on hypercholesterolaemia.

	LRTI without COPD	COPD Exacerbation	
**variable**	missing % (N)	missing % (N)	P value
**aLTRD presentation**	0.0% (0/5994)	0.0% (0/2502)	1
**Age Adjusted CURB Category**	0.0% (0/5994)	0.0% (0/2502)	1
**Age**	0.0% (0/5994)	0.0% (0/2502)	1
**Gender**	0.0% (0/5994)	0.0% (0/2502)	1
**Ethnicity**	0.0% (2/5994)	0.0% (0/2502)	1
**BMI**	76.6% (4590/5994)	75.6% (1891/2502)	0.33
**CCI Category Ex COPD**	0.1% (3/5994)	0.0% (0/2502)	0.56
**IMD (decile)**	1.2% (71/5994)	1.1% (27/2502)	0.74
**Smoker**	0.0% (0/5994)	0.0% (0/2502)	1
**Hypertension**	0.0% (0/5994)	0.0% (0/2502)	1
**AF**	0.0% (0/5994)	0.0% (0/2502)	1
**CVA/TIA**	0.0% (0/5994)	0.0% (0/2502)	1
**IHD**	0.0% (0/5994)	0.0% (0/2502)	1
**CCF**	0.0% (0/5994)	0.0% (0/2502)	1
**Diabetes Type**	0.0% (0/5994)	0.0% (0/2502)	1
**Periph Vasc Dx**	0.0% (0/5994)	0.0% (0/2502)	1
**CKD**	0.0% (0/5994)	0.0% (0/2502)	1
**Troponin Level**	0.0% (0/5994)	0.0% (0/2502)	1

**CRP Level**	0.0% (0/5994)	0.0% (0/2502)	1
**White Cell Count Level**	0.0% (0/5994)	0.0% (0/2502)	1
**Death Within 30 Days**	0.0% (0/5994)	0.0% (0/2502)	1
More than 10% of data is missing for variables BMI.			

Ethics and Permission

Informed consent was obtained from patients with capacity, and declarations for participation from consultees for individuals lacking capacity. Patients who declined consent were not included in this analysis. If it was not practical to approach individuals for consent, data were included using approval from the Clinical Advisory Group (CAG) under Section 251 of the 2006 NHS Act.

Sensitivity Analyses

We performed a sensitivity analysis looking at a biochemical outcome of elevation of high-sensitivity cardiac troponin >18ng/L per Beckman assay to determine if clinical index of suspicion for ACS was equal in both populations. We compared the rate of community prescription of medications which may influence ACS risk (statin, antiplatelet and beta blocker) in patients with and without COPD, in a matched subset of our cohort. We conducted a further sensitivity analysis comparing ACS outcomes excluding patients with asthma (major subgroup in non-COPD control group) to ensure this did not impact our results.

RESULTS

TABLE S2: Respiratory comorbidities in aLRTD patients with and without AECOPD

		**aLRTD without COPD**	**COPD Exacerbation**	
**Variable**	Characteristic	Value (N=5994)	Value (N=2502)	P value
**Asthma**	yes % (n)	16.2% (969)	6.6% (166)	<0.001 †
**Bronchiectasis**	yes % (n)	3.6% (218)	6.6% (164)	<0.001 †
**Chronic Pleural Dx**	yes % (n)	0.3% (17)	0.4% (11)	0.3 †
**Cystic Fibrosis**	yes % (n)	0.1% (8)	0.0% (1)	0.3 †
**Interstitial Lung Dx**	yes % (n)	3.4% (203)	2.6% (65)	0.066 †

"†, Fisher's exact test (categorical) An adjusted P value of 0.01 may be considered significant."

Table S3: Logistic regression models of ACS during admission excluding Asthmatic patients.

		Unadjusted		Adjusted model 1		Adjusted model 2	
**Characteristic**	Group	OR	P-value	OR	P-value	OR	P-value
**COPD LRTI Status**	LRTI without COPD	ref	0.854	ref	0.963	—	—
	COPD Exacerbation	0.94 [0.47–1.87]		1.02 [0.49–2.13]		—	
**COPD Pn Interaction**	No COPD/NP-LRTI	ref	0.119	—	—	ref	0.167
	No COPD/Pneumonia	1.81 [1.05–3.11]		—		1.71 [0.99–2.98]	
	AECOPD/NP-LRTI	1.83 [0.71–4.70]		—		1.70 [0.65–4.40]	
	AECOPD/Pneumonia	0.94 [0.32–2.82]		—		0.85 [0.28–2.56]	
**Age Adjusted CURB Category**	0-1 (Mild)	ref	0.737	—	—	ref	0.709
	2 (Moderate)	1.20 [0.36–3.99]		—		1.15 [0.34–3.88]	
	3-5 (Severe)	0.00 [0.00–Inf]		—		0.00 [0.00–Inf]	
**Age In Decades**		1.13 [0.96–1.32]	0.142	1.12 [0.94–1.33]	0.203	0.90 [0.70–1.16]	0.428
**CCF**	yes	1.83 [0.91–3.70]	0.112	1.80 [0.86–3.77]	0.14	1.45 [0.67–3.12]	0.359
**Gender**	Female	ref	0.087	—	—	—	—
	Male	0.64 [0.38–1.08]		—		—	
**Diabetes Type**	None	ref	0.121	ref	0.117	—	—
	Type 1	0.00 [0.00–Inf]		0.00 [0.00–Inf]		—	
	Type 2	1.54 [0.89–2.66]		1.60 [0.92–2.79]		—	
**Periph Vasc Dx**	yes	1.66 [0.38–7.29]	0.53	—	—	—	—
**CKD**	None	ref	0.365	—	—	—	—
	Mild (CKD 1-3)	0.94 [0.48–1.82]		—		—	
	Moderate or Severe CKD (CKD 4+)	2.32 [0.78–6.88]		—		—	
**CCI Category Ex COPD**	none (0)	ref	0.039	—	—	ref	0.095
	mild (1-2)	3.58 [0.82–15.67]		—		3.91 [0.86–17.69]	
	moderate (3-4)	4.80 [1.12–20.57]		—		6.20 [1.23–31.17]	
	severe (5+)	5.06 [1.18–21.58]		—		6.55 [1.17–36.78]	
**CRP Level**	Unknown	0.00 [0.00–Inf]	0.66	—	—	—	—
	<10	ref		—		—	
	10-50	1.23 [0.66–2.29]		—		—	

	>50	1.12 [0.61–2.05]		—		—	
**White Cell Count Level**	Unknown	0.00 [0.00–Inf]	0.452	—	—	0.00 [0.00–Inf]	0.363
	≤10	ref		—		ref	
	>10	0.79 [0.49–1.27]		—		0.74 [0.45–1.21]	
**IMD (decile)**		0.97 [0.89–1.05]	0.41	—	—	—	—
**Smoker**	Non-smoker	ref	0.575	ref	0.556	—	—
	Current	0.73 [0.32–1.69]		0.85 [0.35–2.06]		—	
	Ex-smoker	0.74 [0.44–1.26]		0.70 [0.40–1.21]		—	
	Unknown	0.59 [0.20–1.69]		0.60 [0.21–1.76]		—	
**Hypertension**	yes	0.68 [0.29–1.61]	0.358	0.55 [0.23–1.34]	0.158	—	—
**AF**	yes	1.05 [0.51–2.17]	0.889	—	—	—	—
**CVA/TIA**	yes	1.05 [0.44–2.48]	0.919	—	—	—	—
**IHD**	yes	0.92 [0.41–2.06]	0.845	0.90 [0.39–2.06]	0.799	0.73 [0.32–1.66]	0.434

Table S4: Relationship between reported acute coronary syndome (ACS) and Troponin levels during admission, in both COPD and non-COPD patients

		LRTI without COPD	COPD Exacerbation
ACS	Cardiac Troponin Level	Count [%] (N=5994)	Count [%] (N=2502)
no	≤18	961/5577 [17.2%]	338/2312 [14.6%]
	>18	1162/5577 [20.8%]	463/2312 [20.0%]
	Unknown	3454/5577 [61.9%]	1511/2312 [65.4%]
yes	≤18	40/417 [9.6%]	18/190 [9.5%]
	>18	191/417 [45.8%]	67/190 [35.3%]
	Unknown	186/417 [44.6%]	105/190 [55.3%]

ACS, acute coronary syndrome; COPD, chronic obstructive pulmonary disease; LRTI, lower respiratory tract infection

TABLE S5: Logistic regression models of elevated troponin during admission.

		Unadjusted		Adjusted model 1		Adjusted model 2	
**Characteristic**	Group	OR	P-value	OR	P-value	OR	P-value
**COPD LRTI Status**	LRTI without COPD	ref	0.159	ref	0.57	—	—
	COPD Exacerbation	0.92 [0.82–1.03]		0.96 [0.85–1.10]		—	
**COPD Pn Interaction**	No COPD/NP-LRTI	ref	<0.001	—	—	ref	<0.001
	No COPD/Pneumonia	1.57 [1.37–1.79]		—		1.40 [1.22–1.61]	
	AECOPD/NP-LRTI	1.13 [0.94–1.35]		—		1.09 [0.90–1.32]	
	AECOPD/Pneumonia	1.34 [1.13–1.58]		—		1.20 [1.01–1.42]	
**Age Adjusted CURB Category**	0-1 (Mild)	ref	0.006	—	—	ref	0.365
	2 (Moderate)	1.25 [1.01–1.53]		—		1.00 [0.81–1.23]	
	3-5 (Severe)	2.29 [1.22–4.30]		—		1.61 [0.84–3.06]	
**Age In Decades**		1.46 [1.40–1.52]	<0.001	1.39 [1.33–1.46]	<0.001	1.24 [1.17–1.31]	<0.001
**CCF**	yes	2.07 [1.81–2.36]	<0.001	1.43 [1.24–1.65]	<0.001	1.48 [1.28–1.71]	<0.001
**Gender**	Female	ref	<0.001	—	—	—	—
	Male	1.30 [1.17–1.44]		—		—	
**Diabetes Type**	None	ref	<0.001	ref	<0.001	—	—
	Type 1	1.63 [1.05–2.52]		2.07 [1.31–3.27]		—	
	Type 2	1.43 [1.26–1.61]		1.31 [1.15–1.48]		—	
**Periph Vasc Dx**	yes	1.60 [1.24–2.06]	<0.001	—	—	—	—
**CKD**	None	ref	<0.001	—	—	—	—
	Mild (CKD 1-3)	1.73 [1.54–1.95]		—		—	
	Moderate or Severe CKD (CKD 4+)	2.28 [1.81–2.88]		—		—	
**CCI Category Ex COPD**	none (0)	ref	<0.001	—	—	ref	<0.001
	mild (1-2)	1.89 [1.19–3.00]		—		1.32 [0.82–2.12]	
	moderate (3-4)	5.08 [3.28–7.87]		—		2.28 [1.41–3.68]	
	severe (5+)	7.21 [4.67–11.15]		—		2.37 [1.44–3.89]	
**CRP Level**	Unknown	0.85 [0.50–1.47]	0.259	—	—	—	—
	<10	ref		—		—	
	10-50	1.07 [0.93–1.24]		—		—	
	>50	0.96 [0.84–1.10]		—		—	
**White Cell**	Unknown	0.35 [0.12–	0.016	—	—	0.41 [0.14–	0.106

**Count Level**		0.98]				1.17]	
	≤10	ref		—		ref	
	>10	1.08 [0.98–1.20]		—		1.05 [0.94–1.17]	
**IMD (decile)**		1.03 [1.01–1.04]	0.005	—	—	—	—
**Smoker**	Non-smoker	ref	<0.001	ref	0.609	—	—
	Current	0.61 [0.50–0.74]		0.91 [0.73–1.13]		—	
	Ex-smoker	1.06 [0.95–1.19]		1.03 [0.90–1.16]		—	
	Unknown	1.08 [0.90–1.31]		1.06 [0.87–1.29]		—	
**Hypertension**	yes	1.74 [1.52–1.99]	<0.001	1.20 [1.05–1.39]	0.011	—	—
**AF**	yes	1.68 [1.48–1.90]	<0.001	—	—	—	—
**CVA/TIA**	yes	1.29 [1.11–1.51]	0.001	—	—	—	—
**IHD**	yes	2.12 [1.85–2.42]	<0.001	1.60 [1.39–1.84]	<0.001	1.65 [1.43–1.89]	<0.001

Table S6: Poisson regression sensitivity analysis

		Unadjusted		Adjusted model 1		Adjusted model 2	
**Characteristic**	Group	RR	P-value	RR	P-value	RR	P-value
**COPD LRTI Status**	LRTI without COPD	ref	0.302	ref	0.201	—	—
	COPD Exacerbation	1.09 [0.93–1.29]		1.13 [0.94–1.35]		—	
**COPD Pn Interaction**	No COPD/NP-LRTI	ref	<0.001	—	—	ref	0.004
	No COPD/Pneumonia	1.59 [1.29–1.96]		—		1.45 [1.17–1.79]	
	AECOPD/NP-LRTI	1.40 [1.06–1.85]		—		1.35 [1.02–1.79]	
	AECOPD/Pneumonia	1.58 [1.22–2.03]		—		1.42 [1.10–1.84]	
**Age Adjusted CURB Category**	0-1 (Mild)	ref	0.004	—	—	ref	0.05
	2 (Moderate)	1.53 [1.17–2.01]		—		1.37 [1.04–1.80]	
	3-5 (Severe)	2.12 [0.98–4.61]		—		1.70 [0.78–3.70]	
**Age In Decades**		1.17 [1.10–1.23]	<0.001	1.14 [1.07–1.21]	<0.001	1.04 [0.96–1.14]	0.338
**CCF**	yes	1.28 [1.04–1.57]	0.021	1.07 [0.86–1.32]	0.54	1.02 [0.82–1.27]	0.85
**Gender**	Female	ref	0.7	—	—	—	—
	Male	1.03 [0.88–1.20]		—		—	
**Diabetes Type**	None	ref	0.709	ref	0.844	—	—
	Type 1	1.17 [0.60–2.30]		1.22 [0.62–2.40]		—	
	Type 2	1.07 [0.89–1.29]		1.02 [0.84–1.23]		—	
**Periph Vasc Dx**	yes	1.22 [0.83–1.80]	0.319	—	—	—	—
**CKD**	None	ref	0.705	—	—	—	—
	Mild (CKD 1-3)	1.07 [0.89–1.28]		—		—	
	Moderate or Severe CKD (CKD 4+)	0.92 [0.61–1.39]		—		—	
**CCI Category Ex COPD**	none (0)	ref	<0.001	—	—	ref	0.03
	mild (1-2)	1.99 [1.08–3.65]		—		1.72 [0.92–3.21]	
	moderate (3-4)	2.39 [1.33–4.30]		—		1.77 [0.92–3.39]	
	severe (5+)	3.34 [1.87–5.96]		—		2.22 [1.13–4.35]	
**CRP Level**	Unknown	0.73 [0.28–1.92]	0.297	—	—	—	—
	<10	ref		—		—	
	10-50	1.18 [0.94–1.48]		—		—	
	>50	1.18 [0.96–1.46]		—		—	
**White Cell Count Level**	Unknown	0.36 [0.05–2.42]	<0.001	—	—	0.40 [0.06–2.67]	0.004
	≤10	ref		—		ref	

	>10	1.34 [1.14–1.58]		—		1.28 [1.09–1.51]	
**IMD (decile)**		1.00 [0.97–1.02]	0.767	—	—	—	—
**Smoker**	Non-smoker	ref	0.059	ref	0.163	—	—
	Current	0.87 [0.66–1.15]		0.95 [0.70–1.29]		—	
	Ex-smoker	1.06 [0.89–1.27]		0.98 [0.81–1.18]		—	
	Unknown	1.35 [1.04–1.76]		1.31 [1.00–1.70]		—	
**Hypertension**	yes	1.22 [0.99–1.49]	0.064	1.00 [0.81–1.23]	0.983	—	—
**AF**	yes	0.98 [0.80–1.20]	0.836	—	—	—	—
**CVA/TIA**	yes	1.08 [0.85–1.37]	0.539	—	—	—	—
**IHD**	yes	1.73 [1.44–2.08]	<0.001	1.58 [1.31–1.92]	<0.001	1.53 [1.26–1.85]	<0.001

TABLE S7: Logistic regression models of elevated troponin during admission.

		Unadjusted	Adjusted model	Adjusted model

				1		2	
**Characteristic**	Group	OR	P-value	OR	P-value	OR	P-value
**COPD LRTI Status**	LRTI without COPD	ref	0.02	ref	0.018	—	—
	COPD Exacerbation	1.43 [1.06–1.93]		1.52 [1.07–2.14]		—	
**Age Adjusted CURB Category**	0-1 (Mild)	ref	0.671	—	—	ref	0.564
	2 (Moderate)	0.82 [0.38–1.77]		—		0.73 [0.33–1.60]	
	3-5 (Severe)	2.33 [0.29–19.04]		—		2.21 [0.27–18.18]	
**Age In Decades**		1.15 [1.03–1.28]	0.01	1.10 [0.98–1.24]	0.108	1.03 [0.87–1.22]	0.731
**Gender**	Female	ref	0.816	—	—	—	—
	Male	1.04 [0.77–1.39]		—		—	
**Diabetes Type**	None	ref	0.483	ref	0.598	—	—
	Type 1	0.43 [0.06–3.14]		0.43 [0.06–3.17]		—	
	Type 2	1.13 [0.79–1.62]		1.06 [0.74–1.52]		—	
**CCI Category Ex COPD**	none (0)	ref	0.005	—	—	ref	0.229
	mild (1-2)	2.63 [0.92–7.55]		—		2.33 [0.79–6.87]	
	moderate (3-4)	3.83 [1.38–10.59]		—		3.04 [0.96–9.62]	
	severe (5+)	3.82 [1.39–10.53]		—		2.91 [0.87–9.70]	
**Periph Vasc Dx**	yes	1.11 [0.48–2.57]	0.812	—	—	—	—
**CKD**	None	ref	0.658	—	—	—	—
	Mild (CKD 1-3)	0.89 [0.61–1.31]		—		—	
	Moderate or Severe CKD (CKD 4+)	1.26 [0.62–2.53]		—		—	
**CRP Level**	Unknown	0.60 [0.14–2.53]	0.702	—	—	—	—
	<10	ref		—		—	
	10-50	1.02 [0.71–1.46]		—		—	
	>50	0.86 [0.59–1.26]		—		—	
**IMD (decile)**		0.98 [0.94–1.04]	0.536	—	—	—	—
**Smoker**	Non-smoker	ref	0.101	ref	0.167	—	—
	Current	0.99 [0.60–1.65]		0.85 [0.48–1.50]		—	
	Ex-smoker	1.23 [0.87–1.74]		0.98 [0.67–1.45]		—	
	Unknown	1.86 [1.13–3.07]		1.65 [0.99–2.75]		—	
**White Cell Count Level**	Unknown	0.78 [0.10–5.81]	0.113	—	—	0.96 [0.13–7.28]	0.257
	≤10	ref		—		ref	
	>10	1.36 [1.01–1.84]		—		1.29 [0.95–1.74]	

**COPD Pn Interaction**	No COPD/NP-LRTI	—	—	—	—	ref	0.081
	No COPD/Pneumonia	—		—		ref	
	AECOPD/NP-LRTI	—		—		1.32 [0.97–1.79]	
	AECOPD/Pneumonia	—		—		ref	
**Hypertension**	yes	1.52 [1.05–2.21]	0.033	1.31 [0.88–1.94]	0.192	—	—
**AF**	yes	1.03 [0.70–1.52]	0.88	—	—	—	—
**CVA/TIA**	yes	1.02 [0.63–1.64]	0.944	—	—	—	—
**IHD**	yes	1.62 [1.12–2.35]	0.014	1.44 [0.97–2.12]	0.074	1.46 [0.99–2.15]	0.064
**CCF**	yes	1.15 [0.76–1.75]	0.518	0.94 [0.60–1.45]	0.767	0.98 [0.63–1.54]	0.936
